# The Unveiled Triad: Clinical, Radiological and Pathological Insights into Hypersensitivity Pneumonitis

**DOI:** 10.3390/jcm13030797

**Published:** 2024-01-30

**Authors:** Gaetano Rea, Marialuisa Bocchino, Roberta Lieto, Roberta Eufrasia Ledda, Michele D’Alto, Marco Sperandeo, Raffaella Lucci, Patrizio Pasquinelli, Stefano Sanduzzi Zamparelli, Giorgio Bocchini, Tullio Valente, Giacomo Sica

**Affiliations:** 1 Department of Radiology, Monaldi Hospital, Azienda Ospedaliera dei Colli, 80131 Naples, Italy; gaetano.rea71@gmail.com (G.R.); roblieto@gmail.com (R.L.); giorgio.bocchini@ospedalideicolli.it (G.B.); tullio.valente@gmail.com (T.V.); 2 Department of Clinical Medicine and Surgery, Section of Respiratory Diseases, University Federico II, Monaldi Hospital, Azienda Ospedaliera dei Colli, 80131 Naples, Italy; marialuisa.bocchino@gmail.com; 3 Section of Radiology, Unit of Surgical Science, Department of Medicine and Surgery (DiMeC), University of Parma, 43121 Parma, Italy; robertaeufrasia.ledda@unipr.it; 4 Department of Cardiology, University “L. Vanvitelli”, Monaldi Hospital, 80131 Naples, Italy; michele.dalto@ospedalideicolli.it; 5 Interventional Ultrasound Unit, Department of Internal Medicine, IRCCS “Casa Sollievo Della Sofferenza” Hospital, San Giovanni Rotondo, 71013 Foggia, Italy; sperandeomar@gmail.com; 6 Department of Pathology, Monaldi Hospital, Azienda Ospedaliera dei Colli, 80131 Naples, Italy; raffaella.lucci@ospedalideicolli.it; 7 Italian Federation of Pulmonary Fibrosis and Rare Pulmonary Diseases “FIMARP”, 00185 Rome, Italy; patrizio.pasquinelli@libero.it; 8 Department of Pulmonary Diseases, San Camillo-Forlanini Hospital, 00152 Rome, Italy; 9 Department of Translational Medical Sciences, University “L. Vanvitelli”, 80131 Naples, Italy; stefanosanduzzi@gmail.com

**Keywords:** hypersensitivity pneumonitis, high-resolution computed tomography, HRCT, BAL, ILD, idiopathic pulmonary fibrosis, IPF, idiopathic interstitial pneumonia, IIP, artificial intelligence

## Abstract

Hypersensitivity pneumonitis (HP) is a diffuse parenchymal lung disease (DLPD) characterized by complex interstitial lung damage with polymorphic and protean inflammatory aspects affecting lung tissue targets including small airways, the interstitium, alveolar compartments and vascular structures. HP shares clinical and often radiological features with other lung diseases in acute or chronic forms. In its natural temporal evolution, if specific therapy is not initiated promptly, HP leads to progressive fibrotic damage with reduced lung volumes and impaired gas exchange. The prevalence of HP varies considerably worldwide, influenced by factors like imprecise disease classification, diagnostic method limitations for obtaining a confident diagnosis, diagnostic limitations in the correct processing of high-resolution computed tomography (HRCT) radiological parameters, unreliable medical history, diverse geographical conditions, heterogeneous agricultural and industrial practices and occasionally ineffective individual protections regarding occupational exposures and host risk factors. The aim of this review is to present an accurate and detailed 360-degree analysis of HP considering HRCT patterns and the role of the broncho-alveolar lavage (BAL), without neglecting biopsy and anatomopathological aspects and future technological developments that could make the diagnosis of this disease less challenging.

## 1. Unmasking the Complexity: An In-Depth Introduction to Hypersensitivity Pneumonitis

HP is a DPLD characterized by complex interstitial lung damage with polymorphic and protean inflammatory aspects. It affects various lung tissue targets, including small airways, the lung interstitium, the alveolar compartment and vascular structures. HP occurs when susceptible individuals develop an exponential and marked immune response to diverse inhaled environmental antigens, both organic and inorganic, such as proteins from mite excrement (e.g., dermatophaghoides pteronyssinus, euroglyphus maynei); mold spores (e.g., aspergillus fumigatus, alternaria alternata); bacteria (mycobacterium avium-intracellulare complex); fungal antigens (e.g., penicillum notatum); and animal proteins (e.g., Feld d1 cat allergen, can f1 dog allergen) [1]. The histopathological marker of the immune-mediated complex is represented by the lymphocytic and plasmocytic inflammatory infiltrate, accompanied by the formation of non-necrotizing granulomas primarily within the context of small airways.

HP shares clinical and often radiological features with other lung diseases in acute or chronic forms. In its natural temporal evolution, if steroid therapy is not initiated promptly during the initial injury phase, it leads to progressive fibrotic damage with reduced lung volumes and impaired gas exchange. Fibrotic alterations manifest with features such that the fibrotic form, “fibrotic HP”, may be erroneously interpreted as a form of idiopathic pulmonary fibrosis (IPF) or another form of idiopathic interstitial pneumonia (IIP), especially if the history is silent for any detectable offending antigen, with no specific data or deducible information from the patient’s clinical history [2]. The prevalence of HP varies considerably worldwide, influenced by factors like imprecise disease classification, diagnostic method limitations for obtaining a confident diagnosis, diagnostic limitations in the correct processing of high-resolution computed tomography (HRCT) radiological parameters, diverse geographical conditions, heterogeneous agricultural and industrial practices and occasionally ineffective individual protections regarding occupational exposures and host risk factors.

Additionally, the accurate estimated prevalence of HP in the global population remains uncertain, mainly due to the possibility that some patients with mild or moderate clinical disease may go unnoticed or be misdiagnosed in the initial clinical and diagnostic approach [3]. Regarding findings and analyses based on insurance claims, prevalence estimates at one year have been reported between 1.67 and 2.71 per 100,000 in the United States population between 2004 and 2013. As evident from sector analyses and literature data, the percentage of HP among all ILD cases shows significantly variable ranges, ranging from 2% to 47% in analyzed studies and registries [4]. Studies on the incidence of HP are currently limited, except for a relatively extensive study with a general population analysis in the United Kingdom estimating an incidence of HP around 1 case per 100,000 [3]. Furthermore, to further consolidate the intrinsic difficulties related to data collection, there currently seems to be no coherent or standardized epidemiological approach to assess various forms of HP. A significant increase in cases can be observed in individuals exposed during sporadic epidemics and in defined professional contexts [3,4].

## 2. Definition, Epidemiology, the “Transition of Classification”, Clinical and Genetic Characteristics and Pathogenesis: Unraveling the HP Enigma

HP represents a diagnostic challenge, and it can remain undetected for years, transitioning from non-fibrotic forms to progressive fibrotic alterations with clinical and imaging similarities to other fibrosing diseases. Compared to other fibrosing ILDs, it has distinctive features that, if correctly identified, can lead to an early diagnosis. However no conclusive evidence supports the idea that every case of untreated HP advances to the fibrotic stage. Additionally, the administration of corticosteroids does not serve as a preventive measure for fibrosis. HP involves a highly intricate process that is not yet fully understood. Firstly, although affecting different lung targets, it may be accompanied by acute systemic manifestations such as fever with shaking chills, even exceeding 39 °C in acute phases, with asthenia and sometimes weight loss. A detailed clinical history significantly aids in clinical framing, as the pathology results from the inhalation of an offending antigen to which the individual becomes sensitized through repeated stimuli, becoming hyper-responsive. Therefore, the overall clinical-radiological-pathological picture of the disease is consequently determined by a combination of the factors listed above, often enhancing the diagnostic confidence of the multidisciplinary team (MDT) to correctly conclude the diagnostic process. A fundamental aspect that deserves high attention to avoid over-diagnosis leading to over-treatments is that a high number of individuals exposed to an offending antigen develop an antigen-specific immune response, limited to the presence of serum IgG antibodies and an increased number of lymphocytes in the lung, without ever subsequently developing the pulmonary disease. This premise remains crucial for understanding that the host’s response is fundamental to the disease’s development [5,6].

Historically known as “extrinsic allergic alveolitis,” HP was divided into three subcategories: acute, subacute and chronic. The temporal timing for distinguishing these three forms had a rationale manifested from clinical, radiological and anatomopathological viewpoints. It is well known that radiologists and pathologists, as “morphological” specialists, cannot diagnose the etiology of a disease without collaboration with clinical specialists. However, the specific temporal conditions that the disease may assume in some situations can lead to the formulation of a “subacute” form of HRCT, characterized by very subtle centrilobular nodules with “fluffy” or “fuzzy” morphology and accompanying ground-glass opacities (GGO) with a predominant upper lobar distribution. However, considering more extensive and in-depth histopathological knowledge, and concerning the overlap between acute and subacute forms, replacement of the old nomenclature, “acute, subacute, and chronic”, was a necessary step, especially considering the increasing importance of fibrotic forms compared to non-fibrotic forms for current therapeutic implications.

Currently, for better diagnostic and therapeutic definition, HP has been divided into non-fibrotic (NFHP) and fibrotic (FHP) forms [4]. Essentially, compared to the past, the condition remains clear: acute, “non-fibrotic” forms undergo a clear improvement and a frankly favorable and benign course with complete recovery if the offending antigen exposure is eliminated and appropriate treatment with steroid therapy is administered. Conversely, other patients, regardless of their subclass, unfortunately do not benefit from treatment, progressing to the fibrotic form responsible for worsening respiratory failure. This may also occur because they are unresponsive to steroids or due to conditions in which, after triggering the pro-fibrotic trigger, the pathological process becomes unstoppable. For the reasons stated, and especially due to the impossibility of treating advanced forms of HP, recent guidelines have oriented temporal parameters into clinical-functional and especially radio-pathological parameters, differentiating between FHP and NFHP forms based on the predominant presence or absence of radiological fibrosis on HRCT and/or histopathological examination, as well as discriminating their clinical-functional profile. This orientation appears even more correct and justified by the current therapeutic possibilities available for FHP forms, now adequately treatable with anti-fibrotic therapy like that administered to IPF patients (previously administered in few selected conditions and severe cases, off-label), and also for the increasingly convincing transition toward a “pattern-based” rather than a “disease-based” therapy.

Regardless of etiology, HRCT and pathological patterns often share the same “outcome”. The classification into FHP or NFHP appears more objective and is likely, confirmed by future studies, to be more consistently associated with clinical course and prognosis [4]. From a clinical perspective, HP is an extremely heterogeneous, diffuse, infiltrative disease with a multimodal presentation. Symptoms can include chronic cough, initially mild dyspnea, later effort-induced and progressively worsening (FHP > NFHP), accompanied by asthenia and weight loss. The physical examination may reveal mid-basal crepitations, classic “velcro sounds” and/or bronchiolar squeaks.

NFHP has a more frequent presentation in young-adult individuals and sometimes allows for more frequent identification of the responsible antigen when a precise clinical history is obtained, and the patient is compliant in providing a detailed description of their professional or leisure habits. Respiratory function tests may highlight a restrictive pattern in confirmed cases, and BAL generally features increased lymphocytosis (in forms with typical features > 25–30%).

FHP, on the other hand, tends to present in subjects of moderately advanced age, older individuals with the typical appearance of a chronic disease with slow progression and less aggressive evolution than idiopathic forms and some secondary to connective tissue disease (CTD). Identifying the responsible antigen is more challenging, and the functional state is generally more compromised. BAL may reveal a pattern of lower or absent lymphocytosis, and sometimes an increase in neutrophils and eosinophils, characteristic of a worse prognosis phenotype [5].

Various triggers are associated with HP, but in approximately 60% of cases, specific antigens and exposure factors remain unidentified despite thorough clinical history examination. The inducing antigens are broadly classified into five categories: bacteria, fungi, yeast, mycobacteria, animal proteins and chemicals [6].

Various antigens, ranging from microbial proteins to chemicals, trigger a severe immune response in affected organisms. Exposure occurs in diverse environments, including domestic and recreational settings. Surprisingly, seemingly harmless activities like hobbies can induce varying degrees of immune responses. A thorough exploration of the clinical history is crucial, as viral infections can facilitate hypersensitivity to environmental antigens. Tobacco smoke, known for causing severe organ damage, influences immune reactivity, guiding the pathogenic process towards fibrotic disease [1].

It is interesting to investigate why, given the universal and wide distribution of offending antigens, only a limited portion of individuals develop the disease: multiple hypotheses on the pathogenesis have been formulated over time, but one of the most accredited is the “two-hit” hypothesis in which pre-existing genetic susceptibility or environmental factors (i.e., the first hit) increase the risk of developing HP after exposure to the antigen (the second hit). Antigen exposure (inorganic particles are haptens, and together with the host’s proteins, they compose antigens) acts as an inducing factor, and genetic or environmental factors act as risk-promoting factors [3]. As for the genesis of inflammation in HP, it is mediated by both humoral and cellular mechanisms; following antigen exposure and processing by the innate immune system, the inflammatory response is predominantly mediated by T-helper cells and antigen-specific immunoglobulins (IgG), leading to the accumulation of lymphocytes and granuloma formation. Regarding the pathogenesis of fibrosis secondary to HP, it is not completely understood due to the multifactorial expression of phenotypic damage. It is believed that in fibrotic disease, abnormal repair mechanisms, secondary to recurrent alveolar epithelial damage and lesions, lead to fibroblast activation and proliferation, extracellular matrix accumulation and subsequent terminal distortion of the lung architecture with irregular interstitial distortion, including patterns conclusively consistent with Usual Interstitial Pneumonia (UIP) [1].

Regarding the genetic profile, pre-existing genetic conditions such as sporadic allelic forms, including the MUC5B rs35705950 allele, have been associated with increased radiological fibrosis extension, as well as genetic alterations determining telomere shortening, with evolution into early senescence forms, correlated with worse clinical survival and greater radio-pathological aggressiveness. Genes that show analogy in patients with HP and IPF have been highlighted in the literature, as well as genes expressed substantially uniquely in HP. The immunopathological profiles and characteristics associated with fibrotic HP are characterized by an increase in CD4+ T cells and the CD4+/CD8+ ratio, a tendency towards Th2 differentiation, as well as depletion of CD8+ T cells. The increase in Th17 cells after chronic inhalation of aerosolized antigens can contribute to the development of FHP through increased collagen deposition (a feature demonstrated by the protective effect against the disease of both IL-17 genetic deletion and IL-17 antibody depletion) [6]. A curious but interesting aspect of this pathology is that the smoking habit is less prevalent in patients with HP compared to unaffected controls with similar antigenic exposure. When exposed to high levels of offending antigens, smokers exhibit lower levels of specific antibodies against the causative antigen. The mechanism through which the protective effect of smoking manifests is not known, but it is hypothesized that nicotine inhibits the activation of macrophages and the proliferation and function of lymphocytes. Although more common in non-smokers, when it occurs in smokers, HP is unfortunately associated with a more chronic and severe course with higher mortality [6]. In an era in which the MDT has assumed a dominant role over the individual specialized figure, incapable on its own of handling the multifactorial characteristics of extremely complex pathologies with difficult clinical framing such as DPLDs and ILDs, the diagnosis of HP also requires a multidisciplinary approach based on history, clinical examination, comprehensive functional profile, laboratory investigations to exclude additional secondary forms, HRCT chest imaging, BAL and histopathology where necessary. These were the motivations behind both the classification transition and the multidisciplinary approach to HP, mandatory for optimal clinical-diagnostic and therapeutic management.

## 3. Decoding HP: Chest HRCT and Pathology in Diagnostic Assessment

**Figure 1:**
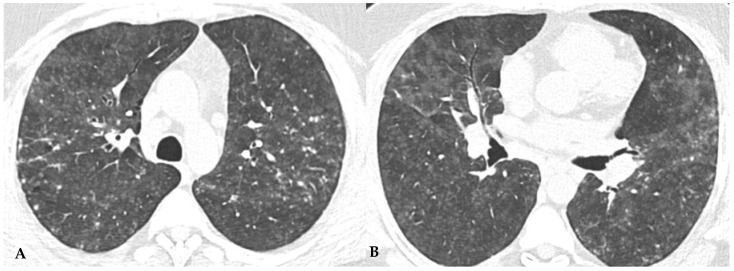

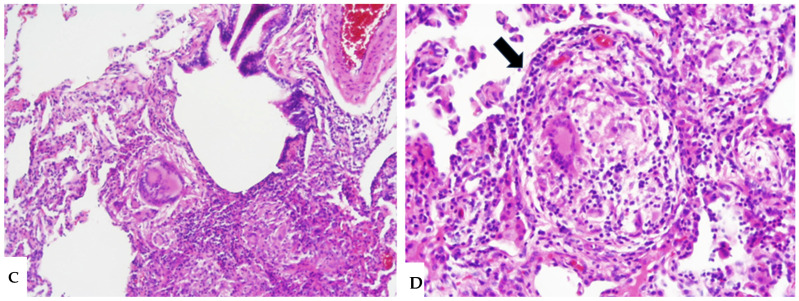
NFHP: axial chest HRCT scan (**A**,**B**): 38-year-old male exposed to molds, inorganic cement dust and glass resin with exertional dyspnea and dry cough: numerous centrilobular “fluffy” nodules with a rosette appearance, suggestive of an acute and non-fibrotic form of hypersensitivity pneumonitis, are observed in both upper lobes and apical segments of the lower lobes. In (**C**,**D**): NFHP: The pathological views after cryo-biopsy (same case (**A**,**B**)) showed airway-centered changes with peribronchiolar damage ((**C**): hem-eo 40×), with interstitial cellular infiltration, poorly formed non-necrotizing granulomas (black arrow) and interstitial giant cells ((**D**): hem. eo 20×). In addition to these findings, areas of GGO may coexist, simulating exudative phases of diffuse alveolar damage (Acute Interstitial Pneumonia, AIP) in the acute phase, along with areas of different lung attenuation called mosaic attenuation (HRCT in inspiration) and air trapping (HRCT in expiration) with “lobular and/or geometric” morphology, indicating acute obstructive damage to small airways (Figure 2).

Due to intrinsic limitations in spatial resolution and contrast, chest X-rays are ineffective in evaluating NFHP, as they may appear normal or demonstrate nonspecific reticular changes in advanced forms. In cases of clinical suspicion, HRCT is considered the gold standard for detecting HP-related alterations. A rigorous methodological approach is required for HRCT to enhance mosaic attenuation areas, which can be accurately diagnosed as “air trapping” in expiratory scans, with a well-known algorithm in the literature: 1/1.25 mm thickness, specific high-resolution convolution kernels and reconstruction filters (bone filters added to soft tissue filters for accurate mediastinal evaluation and assessment of additional ancillary findings) and volumetric acquisition in the inspiratory and expiratory phases (low dose, if necessary). This evaluation plays a key role in diagnosing HP and identifying components that can affect the lungs differently, with heterogeneous characteristics of aggressiveness. Common findings in NFHP reflect inflammation that initially affects small airways with a “bronchiole-centric” type of tropism, analogous to histopathological findings. Lymphoplasmacytic infiltrates (cellular bronchiolitis) with a “fluffy” appearance, leading to the formation of hazy GGO, are often present (Figure 1A,B), along with the production of non-necrotizing granulomas in bronchiolar and peri-bronchiolar locations (Figure 1C,D).

**Figure 2:**
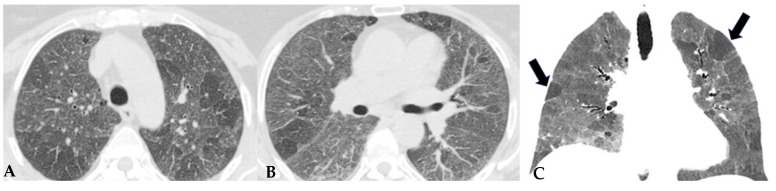
NFHP: chest HRCT ((**A**,**B**): axial scan; C: MPR coronal). GGO pattern and faint centrilobular nodules are observed in both upper lobes and apical segments of the lower lobes (**A**,**B**). Areas of mosaic attenuation (black arrows) are better defined in the coronal MPR reconstruction (**C**). The pattern is suggestive of an acute, non-fibrotic expression of hypersensitivity pneumonitis (NFHP).

**Figure 3:**
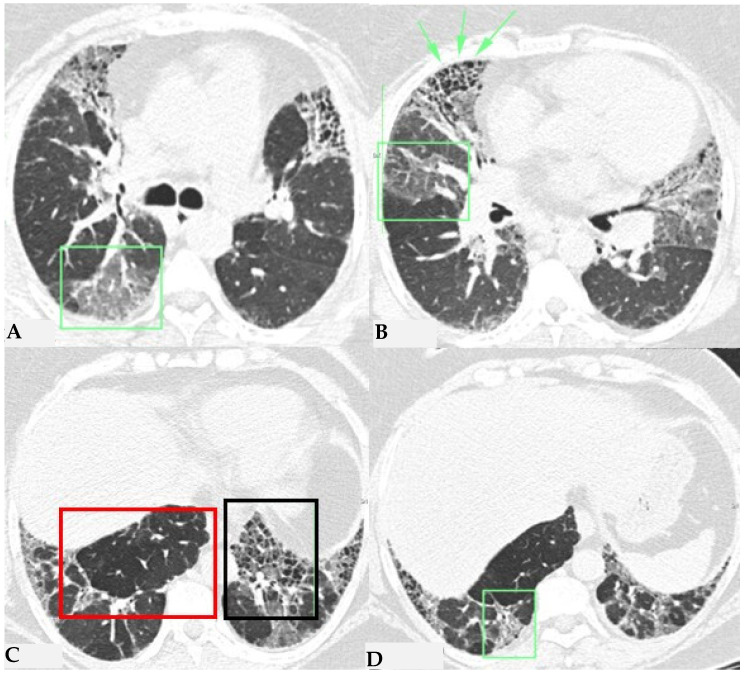
FHP: chest HRCT (**A**–**D**). Upper lobes and right lower lobe are characterized by areas of increased GGO density (green box: (**A**,**D**)) within which well-defined bronchiectasis and traction bronchiolectasis, indicative of established fibrotic damage, are observed (green arrows: (**B**)). Lower lobes extensively affected by mixed alterations, expressing damage to small airways with areas of reduced attenuation suggestive of air trapping (red box: (**C**)), and confirmed fibrotic damage with honeycombing and traction bronchiectasis (black box; (**C**)). Pattern of FHP.

Ancillary findings such as smooth septal thickening and/or pleural effusion may also coexist with typical findings. The presence of numerous inflamed lymph nodes is often described. Another typical finding in the past described as the “head cheese pattern”, now described as “the three-density pattern”, was correlated with the combination of GGO opacities, integrated into a context of air trapping and normal lung. The clear lung heterogeneity, sometimes definitively recognizable in HRCT evaluations, is very suggestive and sometimes pathognomonic of HP in multiplanar reconstructions (Figure 2). Radiological features of HP are influenced by the histopathological stage of the disease at the time of diagnosis. According to international guidelines about HRCT features, HP (both the fibrotic and non-fibrotic forms) can be classified into “Typical HP”, “Compatible with HP” and “Indeterminate for HP”, each presenting typical, compatible or “not-specific” radiological and pathological elements, respectively. HRCT is a pivotal examination for diagnostic confirmation after a comprehensive clinical assessment, functional tests and BAL, with or without transbronchial biopsy (if deemed necessary by the clinician). It is also essential for the ongoing diagnostic process in the follow-up of patients with confirmed diagnoses through a MDT approach, providing information on therapeutic response and defining the possible progression of damage toward fibrotic transition as additional prognostic information useful for evaluating a different therapeutic approach (antifibrotic therapy). The distinctive feature of fibrotic HP is the coexistence of pulmonary fibrosis, often with signs of bronchial obstruction. In fibrotic HP, fibrosis more frequently appears as irregular and coarse reticulation associated with architectural distortion and septal thickening, which may be accompanied by bronchiectasis and bronchioloectasis that is more evident in ground-glass areas (Figure 3), with a centrifugal distribution (broncho-centric) and extension along the bronchovascular axis. These semiotic characteristics significantly differ from the fine and delicate reticulation observed in IPF, reflecting the intrinsic characteristics of immune-mediated inflammatory damage [7,8].

**Figure 4:**
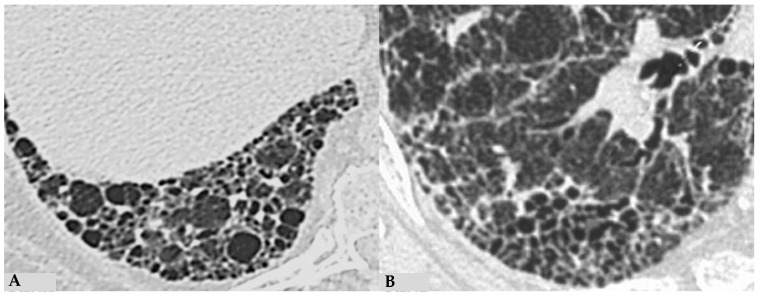
End-stage lung: HRCT magnification of right lower lobes in two different patients. Profuse honeycombing in chronic hypersensitivity pneumonitis (HP) with evidence of multiple multilayered pulmonary cysts ranging in size from a few mm to 10 mm (**A**); honeycombing in idiopathic pulmonary fibrosis (IPF) characterized by minute clustered pulmonary cysts coexisting with subpleural distribution of traction bronchiectasis and bronchiolectasis (**B**).

Similar observations can also be made regarding honeycombing, the terminal process of chronic and progressively realized damage in multiple diffuse lung pathologies. In HP, compared to IPF, honeycombing features gross characteristics, expressed with a “new” term of the semiotic HRCT called “exuberant honeycombing”, with a moderate increase in the size of lung cysts and a radiological UIP pattern. Despite increased knowledge about ILDs, “exuberant honeycombing” in HP remains a diagnostic challenge in the absence of suggestive secondary form history and is also visible in other diseases, for example, CTD-ILDs (Figure 4).

**Figure 5:**
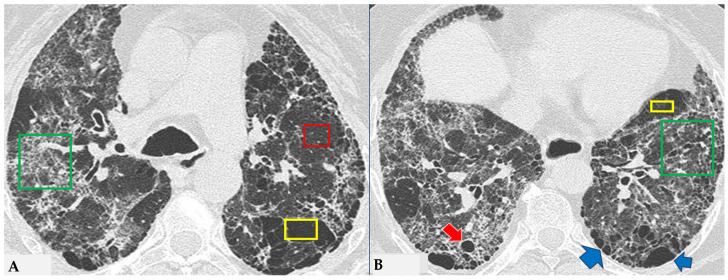
FHP: chest HRCT ((**A**,**B**): axial scan). Visible is the “three density pattern”, with areas of the lung with normal density spared from pathological changes (red box); areas of the lung with reduced density characterized by a paucity of vascular and bronchial structures (yellow box); areas of the lung with increased density showing reticulations, interstitial thickening and traction bronchiectasis (green box). Lung cyst (red arrow) and areas of smoking-related paraseptal emphysema and advanced destructive emphysema (ADE) (blue arrows) are also present.

**Figure 6:**
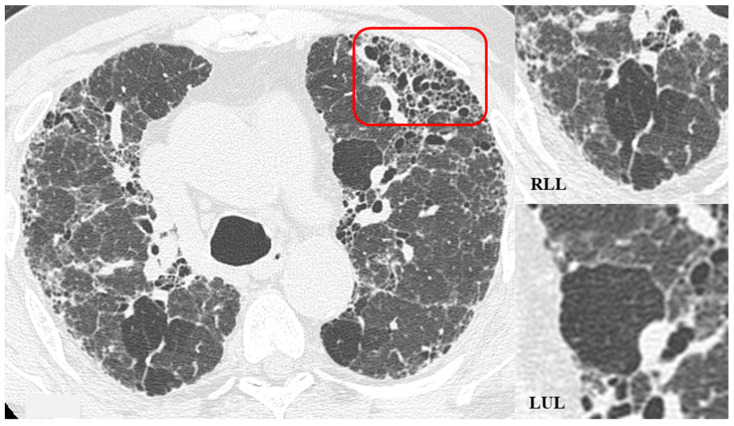
HRCT of the chest and magnification areas of RLL and LUL in FHP. Three-density pattern with lobular areas of reduced attenuation in both lungs (magnification images), overlapping with areas of increased density where distinct fibrotic alterations are evident, characterized by intra- and inter-lobular thickening with traction bronchiectasis and bronchiolectasis (red box). In certain patients, the absence of typical HRCT fibrotic features leads to their classification as “pattern compatible with FHP”. Suggestive elements of this pattern are fibrosis with a mixed central and peripheral distribution (Figure 7) and the fibrotic pattern with basal predominance (Figure 8).

The fibrotic damage in HP is often more severe and extensive in the middle or middle-lower lung zones, but at times, it can be equally distributed across all three lung zones with relative sparing of the subpleural compartment, presenting a “patchy” distribution as a trace of a previous inhalation-mediated immune attack. This distribution may not always exhibit a clear central or peripheral predominance, as evident in axial assessments or MPR reconstructions [7,8,9]. The obstruction of small airways, another significant and distinctive feature of HP, can manifest with various characteristics. In (NFHP), poorly defined centrilobular nodules (fluffy nodules) and mosaic attenuation can be observed. The corresponding HRCT semiotics in FHP presents a “three-density pattern” (Figure 5), characterized by the simultaneous presence of pulmonary lobules with normal density, lobules with (GGO) and lobules with reduced density and size of vascular structures (mosaic attenuation/air trapping) due to air entrapment resulting from broncho-obstruction (Figure 6). The three-density pattern becomes more evident in expiratory HRCT scans due to the air entrapment resulting from the pathogenesis of bronchiole obstruction [4,10].

The absence of characteristic features of a “typical HP pattern” poses the “compatible with HP” pattern as a diagnostic challenge in distinguishing it from some other patterns, including NSIP and atypical forms of UIP/IPF. The clinical presentation can be insidious, characterized by an occasionally silent course, and likely correlated with the absence of a specific acute event of NFHP. Nevertheless, it exhibits non-specific clinical features analogous to other fibrotic ILDs, including dry cough and dyspnea, which may slowly lead to chronic respiratory failure like other idiopathic or secondary forms.

**Figure 9:**
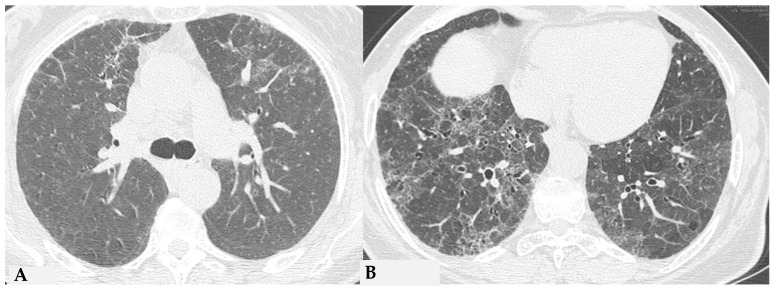
Truly indeterminate HP pattern. Chest HRCT, axial scan HRCT pattern characterized by mild fibrotic changes of a limited extent with patchy GGO in the upper lobes (**A**); the lower lobes show GGO, fine reticulations with modest mosaic attenuation and rare traction bronchiectasis/bronchiolectasis (**B**) with a “truly indeterminate” pattern according to new HP guidelines (2020) [4].

**Figure 10:**
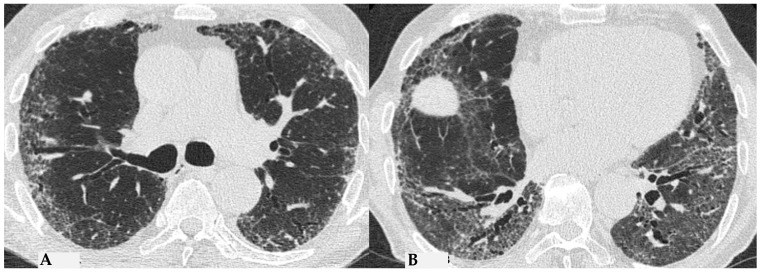
f-NSIP pattern: chest HRCT, axial scan. Mild fibrotic changes of limited extent with a greater distribution in the upper lobes with superimposed GGO (**A**); the lower lobes show marked extension of GGO, fine reticulations with traction bronchiectasis/bronchiolectasis (**B**); “fibrotic NSIP pattern” according to new HP guidelines (2020) [4].

Semeiotic features classified as “indeterminate for FHP pattern” encompass the truly indeterminate HRCT pattern (Figure 9), fibrotic NSIP (Figure 10) and OP. Additionally, due to the primarily bronchocentric nature of the initial damage, the evolution of the fibrotic radiological features may reveal a characteristic centrifugal appearance in MPR reconstructions, with alterations in GGO/consolidative distortive fibrotic changes typically distributed centrally, sometimes sparing the subpleural anatomical surfaces. The patterns outlined in the latest international reference guidelines for HP result from a conclusive diagnostic multidisciplinary approach undertaken in the previous IPF guidelines of 2018 [10]. This correlation addresses the need to introduce a universal language, shared among specialists, that encompasses clinical, radiological and pathological aspects. This ensures the optimal finalization of a diagnostic-therapeutic process for better understanding and simplification [9,10,11]. HP is also coined “the great mimicker”, as it represents a deceptive disease from multiple perspectives; the presence of sometimes hidden antigens makes early diagnosis challenging. The disease course can be deceptive, resembling various pathologies. Without a comprehensive diagnostic panel, including thorough clinical and medical history evaluation, along with functional data and chest HRCT for pattern interpretation, untreated acute cases may progress to severe conditions. This includes the development of fibrotic ILD with end-stage terminal patterns, leading to a secondary but unfavorable outcome of pulmonary fibrosis. In addition to these respiratory complications, pulmonary arterial hypertension (PAH) may develop as a secondary condition to ILD, further compromising the patient’s prognosis. Therefore, it seems appropriate to integrate pneumological and radiological specialist examinations with cardiac evaluations, particularly echocardiography as an initial step, to obtain a diagnostic estimate of pulmonary arterial pressure. This information, in conjunction with complete clinical-functional-diagnostic data, is essential for the treatment of delicate and advanced forms of fibrotic HP with secondary PAH, especially considering the significantly different outcomes of patients with secondary PAH due to FHP [11,12,13,14,15,16].

## 4. Pathologic Features of Fibrotic Hypersensitivity Pneumonitis

**Figure 11:**
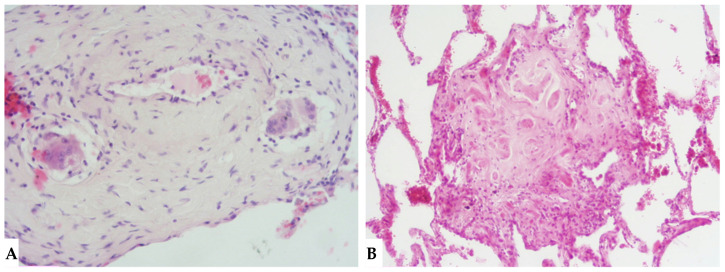
FHP: diffuse fibrotic change with lymphocytic infiltration and giant cells ((**A**): hem-eo 20×); FHP with predominantly airway-centered inflammation characterized by peribronchiolar metaplasia, foamy macrophages in alveolar spaces, poorly formed granulomas and giant cells ((**B**): hem-eo 40×).

**Figure 12:**
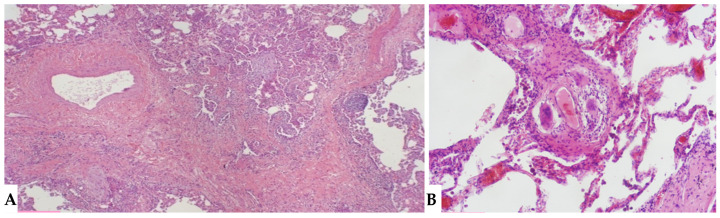
FHP: diffuse fibrotic change with prominent peri-bronchial fibrosis in UIP pattern that is against a diagnosis of UIP/IPF and, according to anamnesis and clinical data, is typical for UIP in FHP (**A**). Schaumann body. Schaumann bodies serve as the equivalent of granuloma tombstones, offering identical diagnostic insight (**B**).

The insidious fibrotic counterpart of HP poses a diagnostic challenge in the ILD scenario from clinical, radiological and purely pathological perspectives. Diagnostic complexities arise partly from the myriad findings discernible in FHP, the divergences regarding defining characteristics of FHP and the chameleon-like nature of FHP to mimic other forms of fibrotic ILDs, particularly UIP/IPF. International guidelines advise against biopsy in clinically and radiologically defined UIP pattern cases. Biopsies are typically reserved for atypical/indeterminate forms. Importantly, a significant proportion of surgical and cryobiopsies in fibrotic ILD contexts correlate with FHP. Distinguishing fibrotic HP from UIP/IPF is not merely an academic exercise, as treatments significantly differ, especially since advanced forms of HP, exhibiting progressive features, do not benefit from corticosteroid treatment but may benefit from antifibrotic therapy. Fibrotic alterations in HP manifest in various forms: bronchiole-centric fibrosis, typically subpleural fibrosis, an NSIP-like fibrotic form and a UIP-like form. Granulomas and giant cells in FHP resemble those in non-HP but, unlike the latter, may exhibit ubiquitous distribution in lung parenchyma rather than being confined to peribronchiolar anatomy. A crucial pathological aspect concerning fibrosis in the evolving framework of HP is the classical “bridging fibrosis” appearance of peribronchiolar metaplasia, typically “air-centered” and better known as “bridging fibrosis”. This feature is generally a reliable pathological marker of FHP, usually affecting a substantial number of bronchioles (>50%). However, occasional foci of peribronchiolar metaplasia may be found in any fibrotic interstitial pneumonia and even in otherwise normal lungs. Therefore, determining the proportion of affected bronchioles is necessary for using this characteristic in FHP diagnosis. Granulomas and giant cells are valuable when present, as are infiltrates and lymphocyte aggregates; however, they are found only in a minority of FHP cases (Figure 11). “End-stage lung” findings with honeycombing indicate a UIP pattern, necessitating correlation with historical, clinical, functional and radiological data, and sometimes pathological data, to provide conclusive information about a specific disease, as isolated findings only signify progressive and final fibrotic damage (Figure 12A). Granulomas/giant cells are similar to those found in NFHP, but unlike the latter, they can be found anywhere in the parenchyma and not just in a peribronchiolar position. Moreover, they are highly useful when present; however, they are found only in a minority of FHP cases. In the differential diagnosis, granulomas and giant cells can occasionally be found in CTD-ILDs or even in other non-necrotizing chronic granulomatous diseases such as sarcoidosis but are not part of pathological alterations seen in UIP/IPF forms [17,18,19]. As in NFHP, Schaumann bodies are occasionally present and serve as substitutes for granulomas (Figure 12B). The interstitial inflammatory infiltrate of FHP may be scanty, as in UIP/IPF, or moderately “cellular,” primarily composed of lymphocytes with a variable number of plasma cells and occasionally some eosinophils when present. Foci of fibroblasts are common in FHP and potentially analogous in pathological configuration to those found in idiopathic forms (UIP/IPF), although often numerically fewer than in idiopathic pathologies such as IPF, and may be associated with isolated peribronchiolar fibrosis. It has also been postulated that fibroblast foci associated with peribronchiolar fibrosis reliably point towards a diagnosis of FHP rather than UIP/IPF diagnosis [20,21]. The differential diagnosis of FHP includes some chronic evolution forms of sarcoidosis, aspiration pneumonitis, collagenopathy-related ILDs, some forms of NSIP and even some clinical-radiological and pathological forms of Interstitial Pneumonia with Autoimmune Features (IPAF). In any case, it is crucial to emphasize that the diagnosis of each ILD is an integrated multidisciplinary process, where each piece must be precisely analyzed by dedicated specialists (pulmonologist, radiologist, pathologist, rheumatologist). Only through the correct interaction of the MDT elements can a coherent and sometimes conclusive diagnosis be achieved.

## 5. BAL: Valuable Test in Respiratory Diagnosis

BAL stands as a helpful diagnostic procedure in the realm of lung pathologies, providing a detailed overview of the physiological state of the respiratory system. Through this technique, cellular samples and fluids are acquired from the junction between the airways and lung alveoli. This rich source of information allows for an in-depth analysis of inflammations, infections and malignancies processes, revealing useful and additional information for defining differential diagnoses and implementing targeted therapies. The diagnostic utility of BAL is particularly relevant in specific lung pathologies, such as HP, sarcoidosis, aspiration pneumonia, lipoid pneumonia, alveolar proteinosis, amyloidosis and also in some cancers. In the context of HP, BAL can unveil the presence of lymphocytic cellular infiltrates with a significant increase in T lymphocytes (>50%), sometimes accompanied by an increase in eosinophils and rarely pigment-laden macrophages, providing a clear indication of a HP-immune response. Conversely, in sarcoidosis, BAL provides information on the characteristic cellular profile of the disease, highlighting the presence of T lymphocytes and activated macrophages with an inverted CD4/CD8 ratio, showing high diagnostic confidence for the disease in the appropriate clinical context. These specific details are crucial for accurate diagnosis and the formulation of targeted therapeutic plans, emphasizing the crucial role of BAL in the context of precision pulmonary medicine. The safety and relative non-invasiveness of this procedure further consolidate its status as a diagnostically reliable tool, significantly contributing to the management and understanding of complex lung pathologies. Interpreting BAL lymphocytosis is not entirely straightforward, as lymphocytosis occurs in various ILDs and can vary widely between fibrotic and non-fibrotic forms of HP. BAL lymphocytosis may be considered a poorly useful parameter when there is a high pre-test probability of HP, as it may not significantly influence the probability of diagnosis, avoiding more invasive tests. However, its utility lies in the potential responsiveness to corticosteroid treatment if radiology is not definitively confident. In this case, a BAL lymphocytosis >30% can increase the overall diagnostic reliability. Such lymphocytosis in a patient with fibrotic ILD is highly suggestive of HP. Still, its absence does not exclude HP, and therefore, lung biopsy must be performed in the appropriate context, considering the patient’s clinical and functional conditions, given its minimally invasive nature and potential to rarely favor disease progression. ATS/JRS/ALAT/JRS guidelines recommend BAL with lymphocytosis evaluation, along with exposure history and HRCT scanning in patients with newly detected ILD before multidisciplinary discussion (MDD). “CHEST” guidelines recommend MDD regarding clinical history and exposures, in addition to HRCT evaluation, before considering diagnostic BAL and not integrating existing diagnostics with a BAL approach if patients present with a history of appropriate clinical context and HRCT pattern typical for HP [4,8,22,23].

### Emergence of Change: Progressive Fibrotic Phenotypes in HP: A New Therapeutic Frontier

**Figure 13:**
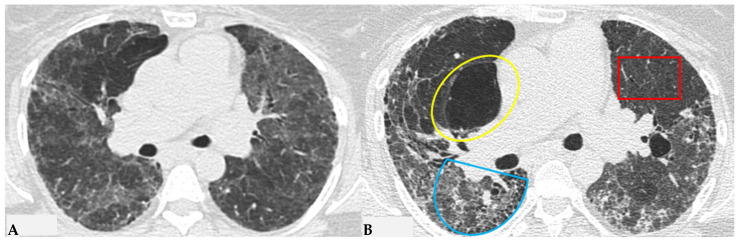
Progressive FHP pattern. Axial chest HRCT scan. Longitudinal assessment of a rapidly progressive form of HP: evident transitions from a cellular form characterized by GGO/centrilobular fluffy nodules and mosaic attenuation (**A**) to a fibrotic form (**B**), characterized by distorted fibrotic GGO, traction bronchiectasis and bronchiolectasis (blue shape), a clear increase in the extensive area of air trapping on the right (yellow circle) and an area of lung spared from the pathological process (red box): a picture of progressive FHP with a “three density pattern”.

HP, as extensively described, represents a diffuse infiltrative disorder caused by sensitization to inhaled antigens that trigger an exuberant and abnormal immune response in the bronchial and alveolar regions of the lungs. Disease susceptibility is regulated by host-related factors, including genetic variations influencing immune response, as well as antigen properties and exposure-related factors. A cluster of patients with fibrotic ILDs, particularly those with FHP and those with CTD-ILDs, show a susceptibility to developing a progressive fibrosing phenotype despite proper therapeutic management: “Progressive Pulmonary Fibrosis” (PPF-ILD) [24,25]. Literature data estimate that approximately 18–32% of patients diagnosed with non-idiopathic fibrotic ILD (non-IPF) may progress towards a progressive phenotype with unfavorable outcomes [26]. This progressive cluster includes different pathologies that share some markers of fibrogenetic activation, showing remarkable similarities with the clinical course observed in aggressive IPF, a prototype of unfavorable prognosis in fibrotic ILDs. Shared pathogenetic mechanisms cause collagen matrix deposition, distorting interstitial architecture and leading to advanced fibrotic damage. This results in a progressive decline in lung function, exacerbated symptoms like dry cough and dyspnea, nutritional deficits and an overall decrease in quality of life. In some cases, this can lead to treatment refractoriness and early mortality. From a genomic perspective, advancements are being made in the search for in vivo biomarkers (liquid biopsy: evaluation of biological fluids such as saliva, sputum, blood, urine, tissues, etc.) with assessment of genomic, proteomic and metabolomic profiles. This would allow for a much more detailed stratification of patients with ILDs in general and those with progressive fibrotic forms in particular [27,28]. This would enable a multimodal cross-sectional approach to understand markers that can further explain aspects of physical decline capable of influencing patient nutrition, thus accelerating physical decline [29]. For this reason, the new aspects related to the decoding of “omics” markers could impact significantly new therapeutic profiles. From a purely radiological standpoint, the new guidelines on IPF and progressive forms make it evident how the role of HRCT evaluation in patients diagnosed with fibrotic ILD can be indispensable in the “timing of serial assessment”, with accurate longitudinal evaluations and side-by-side visual comparison of HRCTs, integrating clinical and functional data and demonstrating possible progression (Figure 13). No standardized protocols exist for treating HP. Prioritizing antigen avoidance is crucial. Though immunosuppressants are frequently employed, their efficacy in slowing fibrotic disease progression remains unproven. The US Food and Drug Administration has sanctioned Nintedanib, a tyrosine kinase inhibitor, for impeding the advancement of chronic fibrosing ILDs, encompassing the progressive fibrotic form of HP. Non-pharmacological measures like oxygen therapy, pulmonary rehabilitation and supportive care play pivotal roles in the comprehensive management of individuals with progressive HP. The new therapeutic frontiers, therefore, open significantly encouraging scenarios in patients with progressive forms, as even these secondary fibrosing forms, previously orphaned of therapy due to their evolutionary characteristics, can finally benefit from antifibrotic therapy like idiopathic forms.

Numerous risk factors influence the progression and mortality of progressive FHP, analogous to other forms of PPF-ILD, including advanced age, male gender, reduced baseline FVC and DLCO, as well as pathological or radiological patterns indicative of UIP characterized by end-stage evolution with traction bronchiectasis and honeycombing consistently represented, along with findings of acute exacerbations.

## 6. Beyond the Surface: Artificial Intelligence’s (AI) Role in Unearthing Hidden Patterns in ILDs Imaging

**Figure 14:**
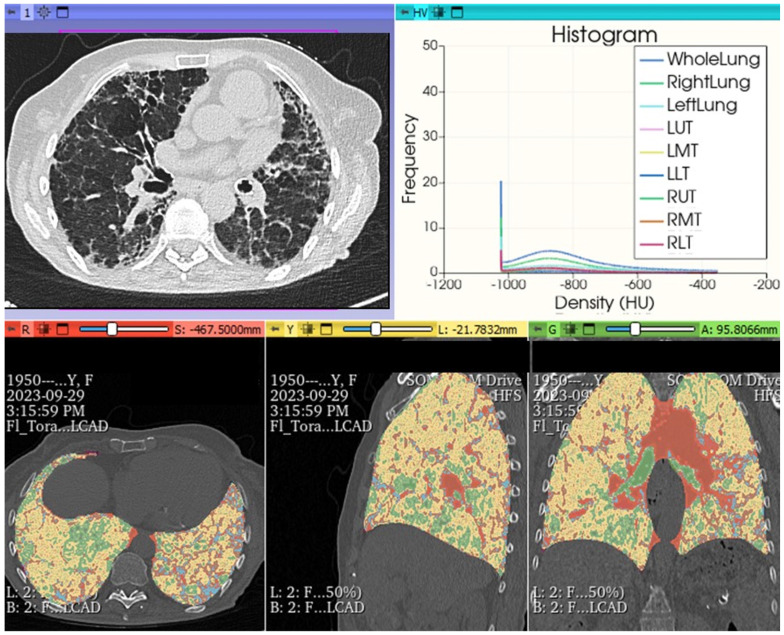
FHP pattern. Chest HRCT: histogram analysis and quantitative reprocessing in MPR on Slicer3D colorimetric scale. AI: radiomics first order features extracted by HRCT after analysis with AI algorithm pyradiomics: whole lungs LAA% −950:13.640; LAA% −910:23.190; HAA% −700:30.560; HAA% −500:16.243; Perc10: −969.000 mean: −719.705; kurtosis: 3.241; skewness: 1.787; ventilation heterogeneity: 0.915; mass 491.189; volume: 1.712; entropy: 2.5032189221693084 energy: 1795731501003.0; variance: 1655.8891640178702; uniformity: 0.18260469191135123.

The growing attention on the evolving profiles of FHP forms correlated with an unfavorable prognosis is further directed towards the radiological imaging realm for a more precise definition of HRCT alterations, as well as therapeutically, with the opening to treatments for progressive forms of HP with anti-fibrotic drugs. The perspective of using both open-source and paid software has, in the last five years, opened horizons previously unimaginable: in ILDs, visual imaging is integrated with quantitative assessments and AI, involving in-depth analysis of HRCT morphological data. Thoracic volume can be extracted, segmented and interpolated with color-coded encodings in post-processing, analyzed comprehensively or for individual lobes of each lung, obtaining conclusive reports rich in numerical and quantitative/radiomic information. This allows for the integration of extremely objective parameters of fibrotic damage associated with clinical and functional data (in vivo biomarkers) and, finally, extrapolating new data to complement classical clinical and functional information. Therefore, a highly sophisticated analysis is possible, introducing classification diagnostic systems with AI, using deep learning algorithms and derived radiomic features. The increasing use of Convolutional Neural Networks (CNNs) for a possible not only diagnostic but also prognostic evaluation offers additional possibilities to explore the complex physio-pathobiological alterations of extremely complex diseases such as ILDs, although currently not yet usable in common clinical practice due to ongoing validation trials concerning the lack of in-depth and robust clinical and control studies (Figure 14). However, in the immediate future, these possibilities will allow for an exceptional approach beyond “visual power”, providing additional potential markers, most of which are not yet known but can further stratify the patient pattern with ILDs, allowing for the analysis of specific “features”, such as radiomics. Today, it is not yet possible to give a specific meaning to these [30,31,32,33,34,35,36,37,38,39].

## 7. Synthesis and Future Visions: Conclusions and Perspectives in HP

The urgent need to deepen our understanding of HP extends across multiple clinical and diagnostic dimensions, implying the unveiling of the intricate complexities of its nature and pathophysiology, advancing diagnostic methodologies, deciphering behavioral patterns and exploring innovative therapeutic modalities. Fundamental investigations into pathophysiology extend to genetic susceptibility, the interplay between host and environmental factors and the subtle differences between predominantly inflammatory and fibrotic subtypes. In the diagnostic domain, crucial requirements include the validation and standardization of questionnaires, with a precise analysis of any exposure factors, BAL, the identification of specific antibodies and the exploration of biomarkers. Further diagnostic investigations such as chest HRCT and the additional evaluation of new techniques like genomic classifiers and AI, including deep learning, radiomics and CNN, are imperative to refine both diagnosis and prognosis. In the clinical suspicion of a patient presenting with an uncertain history, including a persistent cough, dyspnea and pathological objective findings, the pneumologist must necessarily formalize an adequate multidisciplinary protocol to extract a meticulous and complete medical history, potentially using formalized questionnaires to investigate potential temporal correlations between environmental factors/exposures and symptom onset. Additionally, they must integrate laboratory parameters and conduct serum IgG antibody tests against potential HP-associated antigens to identify exposure to potential provocative agents. In the first instance, the clinician should request a HRCT of the thorax, formulating a specific diagnostic question that is directed towards such suspicion, allowing the radiologist to perform the HRCT by integrating any expiratory volumes and increasing diagnostic confidence to perform a correct diagnosis. A range of patients might also require BAL with cellular analysis, with or without transbronchial lung biopsy, if diagnostic confidence levels are not high. In cases where this information, combined with the HRCT pattern, is insufficient for a definitive diagnosis, a MDT is recommended, considering the option of TBBx, TBLC or SLB. This approach is endorsed by ILD experts, based on the best available evidence, subject to review and modification considering evolving evidence. In the field of diagnostic possibilities, the HRCT evaluation is therefore considered the gold standard, being a highly reliable method with extremely high spatial resolution and intrinsic contrast (due to the intra-pulmonary gas component), capable of providing important indications about ILD patterns. Furthermore, thanks to recent and significant technological advancements, especially through quantitative CT analysis and artificial intelligence (including deep learning, radiomics and neural networks), the possibility of expanding with additional in vivo biomarkers to integrate with genomic, proteomic and metabolic data with radiomic data can be increased, raising the qualitative and quantitative level of analysis for each individual patient, with a possible tailored therapy variable from case to case and certainly profiled very accurately. These advanced approaches promise to improve our understanding of ILDs and enhance diagnostic accuracy. HRCT, in the multidisciplinary diagnostic approach, remains a cornerstone in the initial diagnostic evaluation, providing indications on “fibrotic” or “not fibrotic” HP patterns, also allowing, as happens for all ILDs subject to longitudinal evaluations, the interception of temporal evolution variations, defining any progressive phenotypes to allow the clinician to provide the best therapeutic choice.

## Figures and Tables

**Figure 7:**
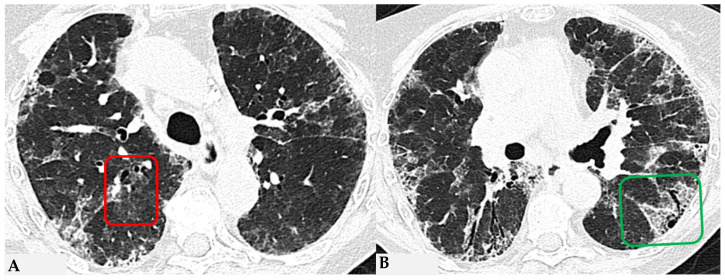
FHP with small areas of GGO related to acute exacerbation: axial chest HRCT scan. HRCT pattern characterized by mild amorphous GGO components in the upper lobes (red box), predominantly centrally located, with sectoral signs of interface and anterior asymmetric reticulations. Minimal traction bronchiolectasis coexists (**A**); subcarinal scan reveals GGO with moderate distortion, septal thickening and alterations predominantly bronchocentric in distribution (green box in (**B**)). The changes exhibit a “patchy” distribution with areas of mosaic attenuation.

**Figure 8:**
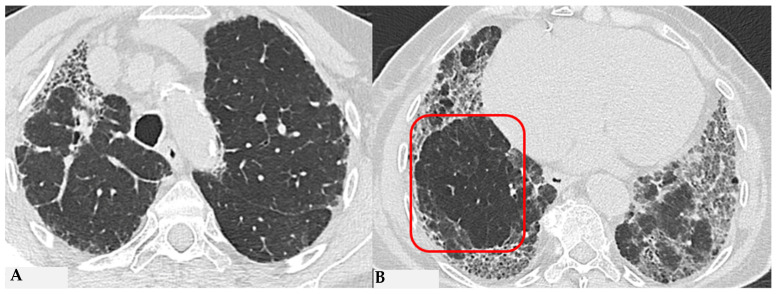
Axial chest HRCT scan. Asymmetric fibrotic alterations with volume loss on the right in the upper lobe (**A**); in the lower lobes on the right, a significant extensive area of reduced attenuation is evident, suggestive of air trapping (red box), with components of distorted GGO and fibrosing characteristics with intra- and interlobular thickening and traction bronchiectasis/bronchiolectasis (**B**).

## Data Availability

Not applicable.
